# Intersecting Molecular Pathways in Cardiovascular Disease and Diabetes Mellitus: Emerging Roles of Inflammation and Therapeutics

**DOI:** 10.1002/dmrr.70167

**Published:** 2026-04-09

**Authors:** Lilian Anagnostopoulou, Nikolaos Ktenopoulos, Anastasios Apostolos, Christos Fragoulis, Panayotis Vlachakis, Paschalis Karakasis, Marios Sagris, Nikias Milaras, Maria Drakopoulou, Andreas Synetos, Ioannis Kyriazis, Ioannis Ioannidis, Costas Tsioufis, Konstantinos Toutouzas

**Affiliations:** ^1^ Internal Medicine Department KAT General Hospital Athens Greece; ^2^ First Department of Cardiology National and Kapodistrian University of Athens Hippokration General Hospital of Athens Athens Greece; ^3^ Second Department of Cardiology Hippokration General Hospital Aristotle University of Thessaloniki Thessaloniki Greece; ^4^ Cardiology Department Hippokration General Hospital of Athens Athens Greece; ^5^ Internist Diabetologist Hygeia Hospital Athens Greece

**Keywords:** cardiovascular disease, diabetes mellitus, diabetic heart disease, gut microbiota, molecular pathways

## Abstract

Diabetes mellitus (DM) and cardiovascular diseases (CVD) remain leading contributors to global morbidity and mortality, imposing a substantial burden on healthcare systems worldwide. The pathophysiological mechanisms underlying these conditions are complex and closely interconnected, with chronic low‐grade inflammation, oxidative stress, endothelial dysfunction, insulin resistance and dysregulated lipid metabolism serving as pivotal shared pathways. Persistent hyperglycaemia and metabolic imbalance in DM accelerate vascular injury and atherosclerotic progression, thereby significantly increasing cardiovascular risk. Consequently, therapeutic strategies that concurrently target both metabolic and cardiovascular dysfunction may offer meaningful clinical advantages and improved long‐term outcomes. In recent years, novel antidiabetic agents such as sodium–glucose co‐transporter 2 (SGLT‐2) inhibitors and glucagon‐like peptide‐1 (GLP‐1) receptor agonists have demonstrated not only glycaemic control but also substantial cardiovascular protection, including reductions in major adverse cardiovascular events, heart failure hospitalisations and renal disease progression. These pleiotropic effects extend beyond glucose lowering and involve modulation of inflammatory pathways, improvement of endothelial function, attenuation of oxidative stress and favourable haemodynamic changes. Additionally, emerging evidence highlights the role of the gut microbiota as a critical mediator in the bidirectional relationship between DM and CVD. Alterations in microbial composition and diversity, collectively termed dysbiosis, have been associated with systemic inflammation, impaired metabolic homoeostasis, increased intestinal permeability and the production of pro‐atherogenic metabolites such as trimethylamine N‐oxide. Understanding these microbiome‐related mechanisms may open new avenues for preventive and therapeutic interventions targeting the gut–metabolic–cardiovascular axis. This narrative review provides an updated and comprehensive overview of the molecular and cellular mechanisms linking DM and CVD, with particular emphasis on inflammatory signalling, metabolic dysregulation and the emerging influence of the gut microbiome in their shared pathogenesis and therapeutic modulation.

## Graphical abstract

Central illustration: Pathophysiological mechanisms linking diabetes mellitus and cardiovascular disease. Chronic hyperglycaemia, insulin resistance, and low‐grade inflammation contribute to endothelial dysfunction, myocardial fibrosis, and cardiomyocyte apoptosis. Key molecular pathways include the nuclear factor‐κB (NF‐κB) activation, oxidative stress, mitochondrial dysfunction, dysregulated calcium handling, and impaired insulin signalling. These interrelated processes culminate in diabetic heart disease, characterised by structural remodelling, diastolic dysfunction, and heightened risk of heart failure. AGEs: Advanced Glycation End‐products, HBP: Hexosamine Biosynthetic Pathway, ROS: Reactive Oxygen Species.

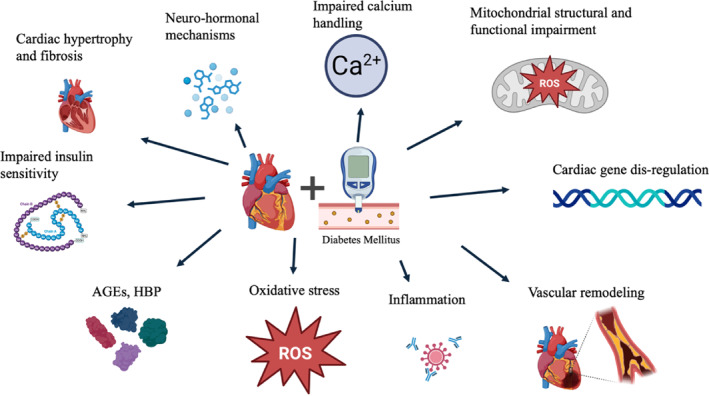

**Abbreviations**
AGEs: Advanced Glycation End Products
Akt: Protein Kinase B
AMPK: AMP‐Activated Protein Kinase
CCR2: C‐C Motif Chemokine Receptor 2
CCR5: C‐C Motif Chemokine Receptor 5
CMR: Cardiac Magnetic Resonance
CV: Cardiovascular
CVD: Cardiovascular Disease
CVOT: Cardiovascular Outcome Trial
DM: Diabetes Mellitus
DPP‐4: Dipeptidyl Peptidase‐4
ECV: Extracellular Volume Fraction
ER: Endoplasmic Reticulum
FABP4: Fatty Acid Binding Protein 4
FGF21: Fibroblast Growth Factor 21
FGFR1: Fibroblast Growth Factor Receptor 1
GLP‐1: Glucagon‐Like Peptide‐1
GLP‐1RA: Glucagon‐Like Peptide‐1 Receptor Agonist
GLS: Global Longitudinal Strain
GLUT4: Glucose Transporter Type 4
HF: Heart Failure
HFpEF: Heart Failure with Preserved Ejection Fraction
HFrEF: Heart Failure with Reduced Ejection Fraction
IGF‐1: Insulin‐Like Growth Factor‐1
IL: Interleukin
MACE: Major Adverse Cardiovascular Events
MI: Myocardial Infarction
NF‐κB: Nuclear Factor Kappa‐Light‐Chain‐Enhancer of Activated B Cells
NLRP3: NOD‐Like Receptor Pyrin Domain‐Containing 3
Nrf2: Nuclear Factor Erythroid 2‐Related Factor 2
PET: Positron Emission Tomography
PI3K: Phosphoinositide 3‐Kinase
PPAR‐γ: Peroxisome Proliferator‐Activated Receptor Gamma
RAAS: Renin–Angiotensin–Aldosterone System
ROS: Reactive Oxygen Species
SGLT‐2: Sodium–Glucose Cotransporter‐2
SGLT‐2i: Sodium–Glucose Cotransporter‐2 Inhibitors
Smad: Small Mothers Against Decapentaplegic Proteins
SPMs: Specialised Pro‐Resolving Mediators
T1D: Type 1 Diabetes
T2DM: Type 2 Diabetes Mellitus
TGF‐β: Transforming Growth Factor Beta
TNF‐α: Tumour Necrosis Factor Alpha
TZD: Thiazolidinedione
VIP: Vasoactive Intestinal Peptide

## 1 Introduction

Cardiovascular diseases (CVDs) and diabetes mellitus (DM) are among the foremost contributors to global morbidity and mortality, posing a significant public health challenge worldwide [1, 2]. The umbrella term ‘cardiovascular disease’ encompasses a variety of conditions, including coronary artery disease, heart failure, stroke, atrial fibrillation and rheumatic or valvular heart diseases. Despite advancements in diagnostics and therapeutic strategies, CVD continues to be the leading cause of death globally [3, 4].

A strong pathophysiological association exists between DM and CVDs (especially those of atherosclerotic origin). Metabolic disturbances characteristic of diabetes, particularly persistent hyperglycaemia and insulin resistance, are recognised triggers of systemic inflammation and oxidative stress, ultimately leading to myocardial and vascular injury [5, 6]. This underlines the importance of comprehensive strategies for prevention, early detection and management of both conditions to improve clinical outcomes and enhance quality of life [7]. Given the mechanistic overlap, pharmacological agents that exert beneficial effects on both DM and CVD are of particular interest. This narrative review explores the molecular mechanisms linking DM to CVD, investigates therapeutic strategies that provide dual benefits and assesses the increasing importance of the gut microbiome [6, 8]. A review of literature was conducted and only articles published in English from 2005 onwards were included, with the most recent search conducted in April 2025 (Central illustration).

## 2 Diabetes Mellitus: Definition and Molecular Mechanisms

### 2.1 Definition and Classification

DM is a heterogeneous group of metabolic disorders characterised by chronic hyperglycaemia due to either impaired insulin production, diminished insulin action, or both [9, 10]. According to the American Diabetes Association (ADA), DM is categorised into four major types: type 1 diabetes (T1D), type 2 diabetes (T2DM), gestational diabetes mellitus (GDM) [11], and specific forms secondary to other conditions. T1D is caused by autoimmune‐mediated destruction of pancreatic β‐cells, resulting in absolute insulin deficiency. Conversely, T2DM is characterised by insulin resistance coupled with progressive β‐cell dysfunction and is often associated with other metabolic abnormalities related to insulin resistance (metabolic syndrome) [12]. Additionally, diabetes can arise secondary to other factors, such as genetic defects in β‐cell function (e.g., MODY), drug‐induced conditions (e.g., corticosteroids), endocrinopathies, or exocrine pancreatic disorders like pancreatitis and haemochromatosis [13]. GDM is diagnosed when hyperglycaemia is first identified during the second or third trimester of pregnancy, in women without a prior history of diabetes [12, 14].

### 2.2 Diagnostic Criteria

The ADA guidelines recommend diagnosing diabetes based on any of the following:

1. Random plasma glucose ≥ 200 mg/dL (11.1 mmol/L) in the presence of symptoms
2. Fasting plasma glucose ≥ 126 mg/dL (7.0 mmol/L) on two occasions
3. 2‐h plasma glucose ≥ 200 mg/dL during a 75 g oral glucose tolerance test
4. HbA1c ≥ 6.5% (48 mmol/mol) [15].

For T1D, the presence of specific autoantibodies, such as anti‐GAD65, IA‐2, anti‐Insulin and anti‐ZnT8 antibodies, supports the diagnosis, even in normoglycaemic stages [16, 17, 18, 19, 20].

### 2.3 Molecular Pathogenesis of Diabetes Mellitus

The molecular pathways linking DM and CVD do not operate as isolated mechanisms but rather form an interconnected network of inflammatory, metabolic and signalling cascades. These pathways can be grouped into functional clusters, including pro‐inflammatory transcriptional hubs, adipokine and hepatokine signalling pathways, lipotoxicity‐related mediators, growth factor and neuropeptide signalling axes, and fibrotic remodelling pathways. These molecular mechanisms converge at the level of specific organ phenotypes such as β‐cell dysfunction, hepatic insulin resistance, endothelial dysfunction and myocardial remodelling (Table 1).

**TABLE 1:** Shared molecular pathways linking diabetes mellitus and cardiovascular disease.

Pathway	Upstream triggers	Key downstream effectors	Metabolic consequences	Cardiovascular manifestations	Therapeutic modulators
Nf‐Κb signalling	Hyperglycaemia, ages, oxidative stress, RAAS activation, gut‐derived metabolites	TNF‐Α, Il‐1β, Il‐6, adhesion molecules	Insulin resistance, chronic inflammation	Endothelial dysfunction, atherosclerosis, myocardial inflammation	Anti‐inflammatory agents, SGLT‐2 inhibitors, GLP‐1 receptor agonists (indirect), experimental Nf‐Κb inhibitors
PI3K/Akt pathway	Insulin receptor activation, IGF‐1 signalling	Akt phosphorylation, GLUT4 translocation	Glucose uptake, metabolic regulation	Cardiomyocyte survival, vascular protection	Insulin sensitizers, metformin, experimental PI3K Modulators
Adiponectin signalling	Adipose tissue endocrine activity	Adipor1/Adipor2 activation, AMPK signalling	Improved insulin sensitivity, reduced inflammation	Anti‐atherogenic effects, improved endothelial function	Weight loss interventions, PPAR‐Γ agonists, experimental adiponectin agonists
FGF21 signalling	Metabolic stress, fasting, mitochondrial dysfunction	Β‐Klotho–FGFR1 signalling	Increased fatty acid oxidation, improved insulin sensitivity	Cardioprotective metabolic effects	FGF21 analogues under investigation
FABP4	Adipose inflammation, lipid overload	PPAR‐Γ modulation, lipid trafficking	Lipotoxicity, insulin resistance	Vascular inflammation, atherosclerosis	FABP4 inhibitors (experimental), metabolic therapies
VIP pathway	Neuroendocrine signalling	Camp signalling, nadph oxidase modulation	Reduced oxidative stress, immune modulation	Improved vascular tone, endothelial protection	Experimental VIP receptor agonists
TGF‐Β/Smad pathway	Chronic inflammation, oxidative stress, RAAS activation	Smad 2/3 signalling	Fibrosis, metabolic dysregulation	Myocardial fibrosis, vascular remodelling	Anti‐fibrotic therapies, RAAS inhibitors

Abbreviations: AGEs: Advanced glycation end products; Akt: Protein kinase B; AMPK: AMP‐activated protein kinase; cAMP: cyclic adenosine monophosphate; CVD: cardiovascular disease; DM: diabetes mellitus; FABP4: fatty acid‐binding protein 4; FGF21: fibroblast growth factor 21; FGFR1: fibroblast growth factor receptor 1; GLP‐1: glucagon‐like peptide‐1; GLUT4: glucose transporter type 4; IGF‐1: insulin‐like growth factor‐1; IL‐1β: interleukin‐1 beta; IL‐6: interleukin‐6; NF‐κB: nuclear factor kappa‐light‐chain‐enhancer of activated B cells; NLRP3: NOD‐like receptor pyrin domain‐containing 3; PI3K: phosphoinositide 3‐kinase; PPAR‐γ: peroxisome proliferator‐activated receptor gamma; RAAS: renin–angiotensin–aldosterone system; ROS: reactive oxygen species; Smad: small mothers against decapentaplegic proteins; TGF‐β: transforming growth factor beta; TNF‐α: tumour necrosis factor alpha; VIP: vasoactive intestinal peptide.

Chronic inflammation is recognised as a key contributor to the pathophysiology of both T1D and T2DM. This inflammatory state results from dysregulation of molecular pathways that govern immune responses and metabolic homoeostasis. The following pathways are central to diabetes‐associated inflammation [21, 22, 23, 24, 25, 26].

- NF‐κB Signalling: Nuclear factor kappa B (NF‐κB) is a pivotal transcription factor involved in the regulation of immune responses and chronic inflammation. Upon activation, NF‐κB induces the transcription of multiple pro‐inflammatory cytokines, including tumour necrosis factor‐alpha (TNF‐α), interleukin‐1 (IL‐1) and interleukin‐6 (IL‐6). Beyond their roles in mediating inflammation, these cytokines also influence cellular processes such as proliferation, differentiation, and survival. Importantly, TNF‐α has been shown to impair insulin signalling by downregulating the expression of glucose transporter type 4 (GLUT‐4) in peripheral tissues. This disruption leads to reduced cellular glucose uptake and contributes to the development of insulin resistance, a key pathogenic feature in type 2 diabetes mellitus [27, 28]. Importantly, NF‐κB signalling represents a key point of convergence for multiple upstream metabolic stressors, including advanced glycation end products (AGEs), renin–angiotensin–aldosterone system activation, gut microbiota–derived metabolites and adipose tissue inflammation. These stimuli frequently converge on inflammatory nodes such as NF‐κB and the NLRP3 inflammasome, amplifying systemic inflammation and cardiometabolic injury.
- Phosphoinositide 3‐kinases PI3K/Akt Pathway: This signalling cascade is critical for cell survival, metabolism, and insulin‐mediated glucose uptake. PI3Ks constitute a family of lipid and protein kinases that play a critical role in regulating cellular growth, metabolism and survival. The PI3K signalling pathway is primarily activated via the insulin receptor and insulin‐like growth factor‐1 receptor (IGF‐1R), leading to the downstream phosphorylation and activation of protein kinase B (Akt/PKB). This cascade promotes cellular survival, proliferation and metabolic homoeostasis. Importantly, PI3K/Akt signalling facilitates the translocation of glucose transporters (GLUTs), particularly GLUT‐4, to the cell membrane, thereby enhancing glucose uptake and improving insulin sensitivity. Impairment of this pathway leads to metabolic dysregulation and defective glucose transport [22, 29].
- Adiponectin Signalling: Adipose tissue, primarily composed of adipocytes, functions as an active endocrine organ by secreting various adipokines, including adiponectin. Adiponectin exerts its biological effects through interaction with its receptors, AdipoR1 and AdipoR2, which can modulate downstream signalling pathways typically associated with insulin receptor activation, notably the phosphoinositide 3‐kinase (PI3K) pathway. Additionally, adiponectin has been shown to influence liver kinase B1 (LKB1) activity, which subsequently modulates NF‐κB signalling. Through these mechanisms, adiponectin may contribute to the attenuation of insulin resistance and exert anti‐atherogenic effects, suggesting a protective role in the pathogenesis of both T2DM and CVD. Impairment of this signalling pathway may diminish adiponectin's insulin‐sensitising and anti‐inflammatory effects, thereby exacerbating glucose intolerance and promoting systemic inflammation, both key features in the development of DM [30].
- FGF21 Pathway: FGF21 has anti‐inflammatory and insulin‐sensitising effects, partly through modulation of macrophage polarisation and subcutaneous adipose tissue storage. Impaired FGF21 activity, whether due to reduced expression, receptor resistance, or downstream signalling defects, leads to dysfunctional adipose tissue, characterised by reduced lipid storage capacity, increased ectopic fat deposition, and a shift towards pro‐inflammatory macrophage phenotypes. These alterations collectively promote insulin resistance, increase circulating free fatty acids and exacerbate systemic inflammation, thereby fuelling the metabolic disturbances that underlie both diabetes and cardiovascular disease [31].
- FABP4: Fatty acid‐binding protein 4 (FABP4), also known as adipocyte FABP or aP2, is a cytoplasmic lipid chaperone predominantly expressed in adipocytes and macrophages. One of its key functions involves the modulation of peroxisome proliferator‐activated receptor gamma (PPAR‐γ) activity, a nuclear receptor essential for adipocyte differentiation and glucose homoeostasis. Through its influence on PPAR‐γ signalling, FABP4 can disrupt normal adipogenic processes and promote lipotoxicity, contributing to insulin resistance. Elevated levels of FABP4 have been associated with metabolic dysfunction and are implicated in the pathophysiology of T2DM, where it exacerbates systemic inflammation and impairs insulin signalling pathways. As such, FABP4 represents both a biomarker and a potential therapeutic target in the management of insulin resistance and related cardiometabolic disorders [32, 33].
- VIP Pathway: Vasoactive intestinal peptide (VIP) is a multifunctional neuropeptide with diverse physiological roles, including the regulation of immune responses, vascular tone and metabolic homoeostasis. One of its notable actions involves the modulation of oxidative stress, primarily through its influence on nicotinamide adenine dinucleotide phosphate (NADPH) oxidase activity. By regulating this enzyme complex, VIP helps to maintain the delicate balance of reactive oxygen species (ROS) production, thereby protecting tissues from oxidative damage. In conditions where VIP expression or activity is diminished, this regulatory mechanism becomes impaired, leading to excessive ROS generation and enhanced oxidative stress, which are key contributors to endothelial dysfunction, inflammation and insulin resistance. These pathological processes collectively promote the onset and progression of T2DM [23].
- The TGF‐β/Smad signalling pathway: contributes to the pathogenesis of both diabetes mellitus and cardiovascular disease by promoting chronic inflammation, fibrosis and metabolic dysregulation. In DM, TGF‐β/Smad impairs insulin signalling, promotes adipose tissue and pancreatic islet fibrosis, and induces β‐cell dysfunction. Concurrently, in the cardiovascular system, it facilitates vascular remodelling, myocardial fibrosis and endothelial dysfunction, thereby contributing to atherosclerosis and diabetic cardiomyopathy. Thus, sustained activation of this pathway serves as a critical molecular link between metabolic and cardiovascular complications [34, 35, 36, 37].

### 2.4 Treatment Strategies

The primary objective in DM management is to maintain an HbA1c at least below 7% (even close to normal with safe agents without risk of hypoglycaemia). However, HbA1c targets should be individualised. Lifestyle modifications, including dietary changes and physical activity [38], are fundamental in T2DM care (Table 2). However, pharmacotherapy is often required [39]:

- Insulin Therapy: Essential in T1D and advanced T2DM [40, 41].
- Biguanides (e.g., Metformin): Enhance insulin sensitivity and promote moderate weight loss [42, 43, 44].
- Thiazolidinediones (TZDs): Improve insulin sensitivity via PPAR‐γ activation [45].
- Depeptidyl peptidase‐ 4 (DPP‐4) Inhibitors (Gliptins): Enhance incretin activity to stimulate insulin secretion [46, 47, 48].
- Glucagon like peptide‐ 1 (GLP‐1) Receptor Agonists: Stimulate insulin release, suppress glucagon, promote satiety, and offer cardiovascular protection [49, 50, 51].
- Dual GLP‐1/Gastric Inhibitory Polypeptide (GIP) Agonists: Enhances glucose‐dependent insulin secretion, suppresses glucagon, delays gastric emptying, promotes satiety, and improves β‐cell function and insulin resistance; leads to HbA_1_C reduction and weight loss with low hypoglycaemia risk [52, 53].
- A next‐generation GLP‐1/GIP/glucagon tri‐agonist: collaboratively boosting insulin release, optimising glucose regulation, and fine‐tuning appetite control, while adding energy expenditure via glucagon receptor activation; achieves HbA_1_C reduction and weight loss, with improved lipids, blood pressure, and albuminuria [54].
- Sodium Glucose Co‐transporter‐ 2 (SGLT‐2) Inhibitors (Gliflozins): Lower plasma glucose by promoting glycosuria, with additional cardio‐renal protective effects [55, 56, 57].
- Ongoing and Emerging Therapies:
  1. Nrf2 activators: Combat oxidative stress.
  2. CCR2/CCR5 antagonists: Target monocyte/macrophage infiltration.
  3. MicroRNA modulation: Experimental therapies modulating miRNAs involved in cardiac remodelling are under investigation.

**TABLE 2:** Pharmacological agents used in diabetes and their cardiovascular, renal and weight effect.

Drug class	Glycaemic effect	Cardiovascular benefit	Weight impact	Renal protection
SGLT‐2 inhibitors	Moderate	++ (HF, CV death)	Mild weight loss	Yes
GLP‐1 receptor agonists	Strong	+ (MACE)	Significant loss	Modest
Metformin	Strong	Possible (secondary)	Mild loss	Uncertain
DPP‐4 inhibitors	Moderate	Neutral	Neutral	Neutral
Insulin	Strong	Neutral	Weight gain	No

Abbreviations: CV: Cardiovascular, DPP‐4: Dipeptidyl Peptidase‐4, GLP‐1: Glucagon‐Like Peptide‐1, HF: Heart Failure, MACE: Major Adverse Cardiovascular Events, SGLT‐2: Sodium‐Glucose Cotransporter‐2.

Notably, metformin, GLP‐1 agonists and SGLT‐2 inhibitors not only improve glycaemic control but also reduce ectopic fat deposition and inflammation in adipose tissue and myocardium [58].

### 2.5 Comorbidities

DM is frequently associated with comorbid conditions that increase cardiovascular risk:

- Hypertension: Present in nearly two‐thirds of diabetic patients due to RAA system activation and sympathetic overactivity [59, 60].
- Obesity: A modifiable risk factor closely linked to insulin resistance [61].
- Polycystic Ovary Syndrome (PCOS): Associated with increased insulin resistance [62].
- Dyslipidaemia: Characterised by elevated triglycerides and VLDL, reduced HDL, and increased oxidative stress [63].
- Obstructive Sleep Apnoea (OSA): Contributes to metabolic dysfunction through hypoxia‐induced sympathetic activation [64, 65, 66].
- MASLD (metabolic dysfunction‐associated steatotic liver disease): Associated with insulin resistance and systemic inflammation [67, 68].

### 2.6 Epidemiology

DM prevalence is escalating globally. In 2013, an estimated 382 million people were affected, increasing to 451 million by 2017. Projections suggest that by 2045, this number will exceed 690 million [69]. This growing burden has profound implications for public health and healthcare systems [28].

## 3 Diabetic Heart Disease

Individuals diagnosed with diabetes mellitus are at a markedly increased risk of developing CVD. Epidemiological studies demonstrate that people with diabetes are approximately twice as likely to experience cardiovascular events, such as myocardial infarction, stroke, and heart failure, compared to non‐diabetic populations. This increased risk applies to both type 1 and type 2 diabetes [70, 71, 72, 73].

Diabetic cardiovascular complications may arise from ischaemic processes, manifest as structural or functional abnormalities of the myocardium (diabetic cardiomyopathy), or involve microvascular and macrovascular damage [74, 75]. Diabetic heart disease (DHD) is specifically defined as myocardial dysfunction occurring in patients with diabetes, independent of concomitant hypertension, coronary artery disease, or other identifiable cardiac pathologies [76, 77, 78, 79].

### 3.1 Functional Impairments That Characterise the Diabetic Heart

DHD is often characterised by subclinical cardiac dysfunction, particularly left ventricular (LV) diastolic dysfunction, which frequently precedes systolic impairment and clinical symptoms [72, 80]. Even in normotensive, asymptomatic individuals with well‐controlled diabetes, a significant proportion exhibit diastolic abnormalities, reflecting early stages of diabetic cardiomyopathy. This dysfunction is associated with impaired myocardial relaxation and delayed LV filling, often in the absence of overt systolic impairment or coronary artery disease. Advances in imaging modalities such as Doppler echocardiography, tissue Doppler imaging and strain‐based techniques (e.g., global longitudinal strain and speckle‐tracking) have enabled the detection of these subtle cardiac abnormalities. Key parameters, including E/A ratio, E/e′ ratio, e′ velocity and isovolumetric relaxation time, are now widely used to evaluate diastolic function [81]. Notably, diastolic dysfunction is a strong predictor of adverse cardiovascular outcomes and may progress to heart failure with preserved ejection fraction (HFpEF) in diabetic patients [82]. These myocardial impairments are considered largely independent of atherosclerosis, as similar changes are observed in diabetic models resistant to coronary artery disease [83, 84, 85, 86]. Thus, early diastolic dysfunction serves as a hallmark of diabetic cardiomyopathy and underscores the need for sensitive diagnostic tools to identify high‐risk individuals before overt heart failure develops [87].

### 3.2 Structural and Microvascular Remodelling in the Diabetic Heart

DM is associated with a spectrum of cardiac structural and microvascular abnormalities that contribute to functional deterioration [72]. These include cardiac fibrosis, hypertrophy, and impaired coronary microcirculation, all of which are increasingly recognised in both clinical and experimental settings [88].

#### 3.2.1 Cardiac Fibrosis

Diabetic cardiomyopathy is characterised by interstitial and perivascular fibrosis, evidenced by elevated myocardial collagen (types I and III) deposition in human biopsy and post‐mortem samples [89, 90]. Non‐invasive imaging modalities such as cardiac magnetic resonance (CMR) with T1 mapping and LGE, as well as echocardiographic backscatter analysis, have confirmed increased fibrosis in diabetic hearts, even in the absence of ischaemia [91, 92, 93, 94]. Fibrosis is linked to adverse cardiovascular outcomes and may represent both reactive and replacement forms [91]. Experimental models corroborate these findings and help elucidate underlying mechanisms.

#### 3.2.2 Cardiac Hypertrophy

Left ventricular (LV) hypertrophy [71]is a structural hallmark of DHD, evident in both sexes and across age groups, with a potential sex‐specific amplification in females [95, 96, 97]. Increased LV mass, detected via echocardiography and CMR, reflects a combination of cardiomyocyte hypertrophy, fibrosis, and myocyte loss. Rodent models of both type 1 and type 2 diabetes frequently exhibit cardiomyocyte hypertrophy [98, 99, 100] although variability exists depending on model type, obesity and insulin resistance, factors that may amplify hyperglycaemia‐induced remodelling.

#### 3.2.3 Coronary Microvascular Dysfunction

Diabetes impairs coronary microvascular perfusion, reducing myocardial blood flow, capillary density and coronary flow reserve, while increasing vascular resistance. Histological analyses reveal rarefaction and arteriolar thickening, with reduced VEGF signalling [101, 102]. These alterations compromise myocardial oxygen and nutrient delivery, promoting fibrotic remodelling and contractile dysfunction. Functional deficits have been observed in both type 1 and type 2 diabetes using PET, CMR, and contrast echocardiography [96, 103, 104]. Animal models mirror these deficits, confirming their relevance to diabetic heart disease pathogenesis.

### 3.3 Advanced Cardiovascular Imaging for the Non‐Invasive Assessment of Diabetic Myocardial Remodelling

Recent advances in cardiovascular imaging have enabled the non‐invasive assessment of myocardial structural and functional changes associated with diabetic heart disease, providing a translational bridge between molecular mechanisms and clinical phenotypes. Speckle‐tracking echocardiography–derived global longitudinal strain (GLS) has emerged as a sensitive marker for detecting subclinical myocardial dysfunction, often preceding detectable reductions in left ventricular ejection fraction in patients with diabetes [105]. In addition, CMR techniques such as native T1 mapping and extracellular volume (ECV) quantification allow for the evaluation of diffuse myocardial fibrosis and extracellular matrix expansion, which are key features of diabetic myocardial remodelling [106]. Furthermore, positron emission tomography (PET) myocardial perfusion imaging provides quantitative assessment of coronary microvascular dysfunction, a hallmark of diabetic cardiomyopathy that reflects impaired endothelial function and metabolic dysregulation. Collectively, these advanced imaging modalities offer powerful tools for linking underlying molecular pathways, such as inflammation, fibrosis signalling, and metabolic stress, to clinically measurable cardiac phenotypes, thereby enhancing the translational relevance of mechanistic insights in diabetic cardiovascular disease [107].

### 3.4 Mechanisms Contributing to Diabetic Heart Disease

A multitude of molecular alterations, including chronic hyperglycaemia, dyslipidaemia, low‐grade inflammation and insulin resistance, contribute to adverse structural and functional changes in the heart and vasculature. These metabolic disturbances activate various pathogenic mechanisms that drive vascular dysfunction and cardiac injury. Long‐recognised contributors include oxidative stress, chronic inflammation, dysregulated calcium homoeostasis, alterations in myocardial substrate metabolism and insulin signalling, transcriptional and epigenetic changes, mitochondrial dysfunction, endoplasmic reticulum (ER) stress, neurohumoral activation, and cardiomyocyte death as referred below [72, 108].

#### 3.4.1 Inflammation and Vascular Remodelling

DM induces a chronic pro‐inflammatory state, driven in part by elevated reactive oxygen species (ROS). A reciprocal relationship between ROS and cytokine signalling creates a feedforward loop that sustains systemic and tissue‐level inflammation [109]. This inflammatory axis is evident in both T1D and T2DM and contributes to multi‐organ complications, including diabetic cardiomyopathy. Increased levels of circulating cytokines (e.g., TNF‐α, IL‐1β, IL‐6), chemokines, and immune cells are observed in patients and rodent models. Diabetic hearts exhibit infiltration of immune cells, particularly pro‐inflammatory (M1‐like) macrophages [110, 111], alongside elevated expression of inflammatory mediators and transcription factors such as NF‐κB. Myocardial inflammation is further amplified by ROS‐mediated activation of the NLRP3 inflammasome, which promotes maturation of IL‐1β and IL‐18, fuelling fibrotic remodelling and LV dysfunction. These mechanisms are confirmed in preclinical models where antioxidant therapy or inflammasome inhibition mitigates cardiac injury. Resolution of inflammation may also be impaired in diabetes. Macrophages show defective efferocytosis, sustaining inflammation [112].

Furthermore, diabetic myocardium often exhibits increased deposition of fibrotic tissue around blood vessels and between myofibrils, independent of overt coronary artery disease or hypertension. Animal models of insulin deficiency show amplified macrophage infiltration in cardiac tissue, along with upregulated expression of adhesion molecules such as ICAM‐1 and VCAM‐1, contributing to leucocyte recruitment and sustained inflammation. Hyperinsulinemia further promotes hepatic production of prothrombotic factors, thereby increasing thrombotic risk [113].

#### 3.4.2 Oxidative Stress

Oxidative stress, defined as an imbalance between excessive reactive oxygen species (ROS) production and impaired antioxidant defence mechanisms, plays a pivotal role in the pathogenesis of diabetic cardiomyopathy. In the diabetic myocardium, ROS overproduction arises primarily from NADPH oxidase activation, mitochondrial electron transport chain dysregulation, and uncoupled nitric oxide synthases [72, 88, 114, 115, 116]. These sources are markedly upregulated under hyperglycaemic and insulin‐resistant conditions. Concurrently, endogenous antioxidant systems, such as superoxide dismutase (SOD), catalase and glutathione peroxidase, are often downregulated, further exacerbating oxidative injury. Preclinical studies in both T1D and T2DM models have demonstrated that pharmacological antioxidants, including SOD mimetics and coenzyme Q10, attenuate myocardial oxidative stress, preventing or reversing cardiac dysfunction. Although clinical evidence remains limited, partly due to suboptimal antioxidant strategies, elevated ROS levels are well‐documented in diabetic human myocardial and vascular tissues [117, 118, 119]. Beyond direct oxidative damage to proteins, lipids, and DNA, ROS also act as signalling molecules, promoting activation of pro‐inflammatory and pro‐apoptotic pathways, including inflammasomes, protein kinase C (PKC), apoptosis signal‐regulating kinase 1 (ASK1), p38 MAPK, JNK, and JAK‐STAT cascades [72, 100, 108, 114, 120, 121, 122, 123]. These pathways not only mediate cardiac injury but also reinforce ROS production, establishing a pathological feedforward loop.

#### 3.4.3 Altered Cardiac Metabolic Pathway

Diabetes disrupts myocardial energy metabolism, characterised by decreased glucose oxidation, enhanced fatty acid oxidation, and reduced glycolysis [108, 124]. Mitochondrial ROS production, including ROS‐induced uncoupling [125, 126], further reduces cardiac efficiency. Additionally, the accumulation of advanced glycation end‐products (AGEs) and increased flux through the hexosamine biosynthesis pathway (HBP) contribute to glucotoxicity [72, 108] and cardiac dysfunction.

##### 3.4.3.1 Advanced Glycation End‐Products (AGEs)

AGEs result from non‐enzymatic glycation of proteins and accumulate under chronic hyperglycaemia. AGE modification of extracellular matrix (ECM) proteins impairs myocardial compliance and promotes fibrosis. Engagement of AGEs with their receptor RAGE amplifies oxidative stress and inflammation, establishing a pathogenic feedforward loop [108, 127]. Pharmacological targeting of this axis (e.g., with Alagebrium or RAGE deletion) has demonstrated cardioprotective effects in preclinical models. AGE accumulation also affects key intracellular proteins such as SERCA2a [128, 129] and sarcomeric components, contributing to impaired calcium handling and contractility. Although clinical trials targeting AGEs have been initiated, most remain incomplete due to non‐efficacy or financial limitations.

##### 3.4.3.2 Hexosamine Biosynthesis Pathway (HBP)

The HBP shunts a small fraction of glucose to produce UDP‐N‐acetylglucosamine (UDP‐GlcNAc), which serves as a substrate for O‐linked N‐acetylglucosamine (O‐GlcNAc) protein modification [130, 131]. In diabetes, sustained hyperglycaemia and oxidative stress enhance HBP flux, increasing protein O‐GlcNAcylation, which modulates numerous proteins involved in calcium handling, metabolism, and inflammatory signalling [132]. Dysregulation of this pathway, including increased GFAT activity and reduced OGA expression, leads to persistent O‐GlcNAcylation in the diabetic myocardium. This modification promotes inflammatory responses, ER stress, and impaired cardiac function [132, 133]. While transient O‐GlcNAcylation may exert protective effects, sustained elevation in diabetes is maladaptive. Cardiac‐targeted interventions reducing O‐GlcNAcylation have shown benefits in rodent models, supporting its pathogenic role in diabetic cardiomyopathy.

#### 3.4.4 Impaired Cardiac Insulin Signalling in Diabetes

Systemic insulin resistance is a hallmark of diabetes and extends to the myocardium, where insulin sensitivity is disrupted at multiple levels. In diabetic hearts, insulin‐stimulated glucose uptake is reduced due to decreased expression and impaired translocation of GLUT4 to the sarcolemma in cardiomyocytes [72]. These changes are accompanied by defects in insulin‐mediated regulation of myocardial contractility and vascular tone, involving impaired signalling through phosphoinositide 3‐kinase (PI3Kα) and its downstream effector, Akt [134].

The relationship between altered insulin signalling and diabetic cardiomyopathy is multifaceted. On one hand, impaired activation of the PI3Kα/Akt pathway may compromise cardioprotective mechanisms and promote cardiac dysfunction. On the other, selective overactivation of alternate insulin signalling branches, including those involving insulin receptor substrate 1 (IRS1) [135, 136] and cross‐talk with β‐adrenergic signalling via G protein‐coupled receptor kinase 2 (GRK2), may contribute to pathological left ventricular (LV) remodelling.

Insulin and insulin‐like growth factor‐1 (IGF‐1) receptor signalling are both altered in diabetes, further disrupting myocardial metabolic and functional homoeostasis. Experimental models have shown that impaired PI3Kα/Akt signalling [100] can exacerbate diabetic cardiomyopathy, whereas targeted enhancement of PI3Kα activity mitigates cardiac oxidative stress and inflammation, offering cardioprotection [137, 138].

#### 3.4.5 Diabetes‐Induced Impairments in the Mitochondrial Structure and Function

Mitochondria play a central role in redox signalling and metabolic regulation [139]. In T2DM, both clinical and experimental evidence show impaired mitochondrial dynamics and excess ROS production, surpassing antioxidant defences and promoting oxidative stress and inflammation [72, 117]. Although mitochondrial number may increase, organelles are smaller and fragmented, reflecting disrupted fission/fusion balance. This is supported by decreased expression of mitofusin‐1 in diabetic myocardium [140]. Such dysfunction contributes critically to the pathogenesis of diabetic cardiomyopathy and is exacerbated by altered substrate metabolism and impaired insulin signalling. Mitochondrial abnormalities mirror cytosolic disturbances seen in hyperglycaemic states.

##### 3.4.5.1 Mitochondrial ROS

Hyperglycaemia and lipid overload drive mitochondrial ROS generation, as observed in diabetic human myocardium [117]. Elevated ROS impairs mitochondrial dynamics and structure, partly via dysregulation of fission/fusion regulators such as DRP1 and OPA1, further aggravating mitochondrial dysfunction in diabetes [141].

##### 3.4.5.2 Mitochondrial O‐GlcNAcylation

Mitochondria contain an intrinsic O‐GlcNAcylation system, with OGT and OGA modulating protein modification via UDP‐GlcNAc imported from the cytosol [142, 143]. Hyperglycaemia enhances O‐GlcNAcylation of respiratory complex subunits (e.g., NDUFA9) and proteins regulating mitochondrial dynamics (e.g., DRP1, OPA1, TFAM, and mitofusin‐2). Although short‐term models show early increases in mitochondrial O‐GlcNAcylation, its long‐term impact on cardiac function and prevalence in T2DM remain unclear. Nevertheless, maladaptive O‐GlcNAcylation may represent a key mechanism linking mitochondrial dysfunction to diabetic cardiomyopathy.

#### 3.4.6 Myocardial Cell Death Pathways

Oxidative and ER stress, along with disrupted mitochondrial dynamics, contribute to cell death in diabetic hearts. Apoptosis, autophagy, and necrosis are elevated in both T1D and T2DM models [72, 101, 108]. While low levels of apoptosis and autophagy are essential for cellular homoeostasis, excessive cardiomyocyte apoptosis and subsequent fibrosis are pathological. The role of autophagy is less clear and may depend on insulin signalling status, with reduced signalling enhancing and excessive signalling suppressing autophagy. Lipotoxicity further disrupts autophagic regulation. Findings from animal models are mixed, some show increased autophagy with mild cardiac remodelling, while others demonstrate impaired autophagic flux [144, 145]. Mst1 may govern the balance between apoptosis and autophagy [146]. Additionally, diabetes impairs endogenous cytoprotective mechanisms, contributing to overall myocardial vulnerability. Subclinical cardiac injury is evident in T2DM and prediabetes, as reflected by elevated troponin levels in epidemiological studies.

#### 3.4.7 Lipotoxicity, Mitochondrial Dysfunction, and ER Stress

Excess fatty acid uptake and oxidation in diabetic hearts imposes metabolic stress, increasing myocardial oxygen consumption and reducing cardiac efficiency [147]. Lipid accumulation within cardiomyocytes, termed lipotoxicity, impairs cellular function and promotes apoptosis. Mechanisms underlying lipotoxic injury include ceramide accumulation, ROS generation, and structural changes in mitochondrial membranes [148, 149]. Endoplasmic reticulum (ER) stress is also implicated, driven by increased saturated lipid content and alterations in mitochondrial phospholipid composition [150].

#### 3.4.8 Impaired Calcium Handling

Delayed Ca^2+^ transient decay is a consistent finding in diabetic cardiomyocytes, often preceding LV systolic dysfunction [95, 151]. This is primarily attributed to reduced SERCA2a expression or activity, potentially due to post‐translational modifications from glycation, O‐GlcNAcylation, or oxidative stress. Broader impairments in Ca^2+^ handling, affecting influx/efflux, SR and mitochondrial Ca^2+^ storage, and key regulators such as phospholamban and ryanodine receptors, are also evident [72, 124, 128, 152]. Additionally, myofibrillar Ca^2+^ responsiveness is diminished. Early studies identified Ca^2+^ dysregulation as a hallmark of diabetic cardiomyopathy. Reduced PI3Kα signalling and inward Ca^2+^ current deficits, as seen in T1D models like the Akita mouse [153, 154] further compromise contractility. Collectively, these abnormalities contribute to impaired cardiomyocyte relengthening and LV diastolic dysfunction in DM.

#### 3.4.9 Neurohormonal Mechanisms

Activation of the renin–angiotensin–aldosterone system (RAAS) and endothelin‐1 signalling is well‐documented in diabetes, both systemically and within the myocardium [72, 155, 156]. RAAS upregulation increases afterload and promotes myocardial remodelling through direct local effects [157, 158]. Pharmacologic and genetic RAAS inhibition attenuates cardiac dysfunction in diabetic models, supporting its role in disease pathogenesis. Accordingly, RAAS blockade (e.g., ACE inhibitors or ARBs) is recommended in heart failure patients with diabetes and preserved renal function. Diabetes is also associated with cardiac autonomic dysfunction, contributing to arrhythmias and heightened myocardial inflammation, although therapeutic strategies to address this remain limited. Additionally, fibroblast growth factor 21 (FGF21) [159], a metabolic regulator elevated in T2DM, promotes fatty acid oxidation and glucose utilisation while suppressing lipogenesis. While FGF21 may exert compensatory cardiometabolic effects, its direct role in diabetic heart disease requires further investigation [160, 161].

#### 3.4.10 Cardiac Hypertrophy—Cardiac Fibrosis

Cardiac hypertrophy arises from chronic metabolic stress, including hyperglycaemia, insulin resistance, and neurohormonal activation, which stimulate pro‐hypertrophic signalling pathways such as MAPK, PKC, and calcineurin/NFAT. This leads to increased cardiomyocyte size and impaired cardiac efficiency. Simultaneously, myocardial fibrosis results from excess extracellular matrix deposition driven by pro‐fibrotic mediators like TGF‐β, angiotensin II, and oxidative stress. Fibrosis increases myocardial stiffness and disrupts diastolic function [71]. Together, these structural changes compromise ventricular compliance and contribute to diastolic dysfunction, a hallmark of diabetic heart disease. Over time, this may progress to heart failure, underscoring the importance of targeting hypertrophy and fibrosis in the management of diabetic cardiomyopathy [98].

#### 3.4.11 Changes at the Level of Cardiac Gene Regulation

Epigenetic mechanisms and non‐coding RNAs, particularly microRNAs (miRNAs), are emerging as important modulators of cardiac remodelling in diabetes [72, 124, 162]. Dysregulated expression of several miRNAs, such as upregulation of miR‐199a/b, miR‐210, miR‐223, and miR‐34 family, and downregulation of miR‐1, miR‐133a, and miR‐203, has been observed in both experimental and human diabetic myocardium. Some miRNAs (e.g., miR‐1, miR‐133a, miR‐203) [162] appear cardioprotective, limiting apoptosis, hypertrophy, and fibrosis, while others may exacerbate pathological remodelling.

Beyond miRNAs, long non‐coding RNAs (lncRNAs) [146] circular RNAs (circRNAs), and small nucleolar RNAs (snoRNAs) are also modulated by hyperglycaemia and may influence cardiac metabolism and stress responses. Additionally, diabetes alters epigenetic marks, including DNA methylation and histone modifications, in both human and animal hearts [163, 164]. Although causality remains to be fully established, these regulatory mechanisms are likely contributors to the development and progression of diabetic cardiomyopathy and represent potential therapeutic targets.

Diabetic cardiomyopathy can progress without noticeable symptoms, underscoring the importance of early detection via echocardiographic and MRI‐related markers. The cardioprotective effects of SGLT‐2 inhibitors and GLP‐1 receptor agonists are, in part, attributed to improvements in myocardial metabolism, inflammation, and structural modifications [165, 166].

## 4 Pharmacological Approaches in Diabetes and Cardiovascular Disease

Standard treatment regimens for diabetes mellitus include a variety of pharmacological agents, often used in combination to achieve optimal glycaemic control. Among these, SGLT‐2 inhibitors and GLP‐1 receptor agonists have demonstrated efficacy not only in lowering glucose levels but also in reducing cardiovascular risk. Metformin, DPP‐4 inhibitors, and insulin are also key pharmacologic agents in DM management, each with distinct effects on cardiovascular outcomes. Table 3 summarises key cardiovascular outcome trials evaluating the cardiovascular effects of major antidiabetic drug classes.

**TABLE 3:** Major cardiovascular outcome trials of antidiabetic therapies and their principal cardiovascular findings.

Drug class	Representative trials	Population	Major cardiovascular findings	Mechanistic interpretation
SGLT‐2 inhibitors	EMPA‐REG OUTCOME, CANVAS, DECLARE‐TIMI 58, DAPA‐HF, EMPEROR trials	T2DM with high CV risk; HF populations with and without diabetes	Reduced hospitalisation for heart failure and CV mortality	Supports mechanisms involving natriuresis, improved cardiac metabolism, reduced inflammation and oxidative stress
GLP‐1 receptor agonists	LEADER, SUSTAIN‐6, REWIND, HARMONY outcomes	T2DM with established or high CV risk	Reduced major adverse cardiovascular events (MACE) including CV death, MI, and stroke	Supports anti‐inflammatory, anti‐atherosclerotic and metabolic effects
DPP‐4 inhibitors	SAVOUR‐TIMI 53, EXAMINE, TECOS, CARMELINA	T2DM with CV disease or high CV risk	Overall cardiovascular neutrality; possible HF signal with saxagliptin	Suggests glycaemic benefit without strong cardioprotective mechanisms
Thiazolidinediones (TZDs)	PROactive, IRIS	T2DM or insulin resistance with CV risk	Mixed results; some reduction in stroke/MI but increased risk of heart failure	Reflects insulin‐sensitising benefits but adverse fluid retention effects
Metformin	UKPDS follow‐up	Overweight T2DM	Reduction in macrovascular complications and mortality	Supports insulin‐sensitising and anti‐inflammatory mechanisms

Abbreviations: CV, cardiovascular; CVOT, cardiovascular outcome trial; DPP‐4, dipeptidyl peptidase‐4; GLP‐1RA, glucagon‐like peptide‐1 receptor agonist; HF, heart failure; HFpEF, heart failure with preserved ejection fraction; HFrEF, heart failure with reduced ejection fraction; MACE, major adverse cardiovascular events; MI, myocardial infarction; SGLT‐2, sodium–glucose cotransporter 2; T2DM, type 2 diabetes mellitus; TZD, thiazolidinedione; UKPDS, United Kingdom Prospective Diabetes Study.

### 4.1 SGLT‐2 Inhibitors

Sodium–glucose co‐transporters (SGLTs), located on the luminal membrane of proximal renal tubules, mediate glucose reabsorption from the glomerular filtrate. SGLT‐2 inhibitors (e.g., empagliflozin, dapagliflozin) are orally administered agents that selectively inhibit SGLT‐2 in the early proximal tubule, reducing glucose reabsorption by 50%–60%, thereby promoting glucosuria and lowering plasma glucose levels [167].

Initially developed as antihyperglycaemic agents for T2DM, SGLT‐2 inhibitors have demonstrated additional cardiovascular and renal benefits. Clinical trials have consistently shown that these agents reduce blood pressure and significantly lower the risk of hospitalisation for heart failure, irrespective of diabetic status, in patients with both reduced and preserved ejection fraction [168].

The cardioprotective mechanisms of SGLT‐2 inhibitors are multifactorial. They include natriuresis and plasma volume contraction, leading to increased haematocrit and reduced preload and afterload. Improvements in endothelial function, attenuation of blood pressure, and modulation of sodium handling also contribute. Additional benefits involve reduced adipose tissue inflammation, decreased pro‐inflammatory cytokine production, diminished oxidative stress, lower serum uric acid, ketone body utilisation by cardiac and renal tissues, and inhibition of AGE‐related signalling pathways [168, 169].

### 4.2 GLP‐1 Receptor Agonists

Glucagon‐like peptide‐1 (GLP‐1) is an incretin hormone that enhances glucose‐dependent insulin secretion and plays a central role in postprandial glucose regulation via the gut–pancreas axis. The ‘incretin effect’ results in greater insulin release following oral versus intravenous glucose administration [170].

Beyond glycaemic control, GLP‐1 receptor (GLP‐1R) activation exerts multiple beneficial effects [171]. It supports pancreatic β‐cell function and survival, reduces inflammation, improves cardiac performance, regulates lipid metabolism, and promotes neurogenesis. GLP‐1 also suppresses appetite and delays gastric emptying, contributing to weight reduction.

GLP‐1 receptor agonists (GLP‐1 RAs), such as semaglutide and liraglutide, have demonstrated cardiometabolic benefits, including blood pressure reduction, improved lipid profiles, and weight loss [172]. These combined effects suggest a meaningful reduction in cardiovascular risk in both diabetic and non‐diabetic populations [173, 174].

### 4.3 Metformin

Metformin remains the first‐line therapy due to its proven efficacy in glycaemic control and cardiovascular benefits. It improves insulin sensitivity and reduces hepatic glucose production. Notably, it has been associated with reduced macrovascular complications and lower cardiovascular mortality, especially in overweight patients [175].

### 4.4 DPP‐4 Inhibitors

DPP‐4 inhibitors (e.g., sitagliptin, linagliptin) enhance endogenous incretin action, promoting glucose‐dependent insulin release. They are weight‐neutral and have a low risk of hypoglycaemia [176, 177, 178, 179]. While generally cardiovascular‐safe, they have not consistently shown significant cardioprotective effects beyond neutrality in major trials, though some agents (like linagliptin) may be preferred in renal impairment [180, 181, 182].

### 4.5 Insulin

Insulin, essential in advanced disease or acute hyperglycaemia, effectively lowers glucose but carries risks of weight gain and hypoglycaemia, which can adversely affect cardiovascular outcomes. While necessary in many cases, intensive insulin therapy has not demonstrated clear cardiovascular benefit and may increase the risk of adverse events if not carefully managed [183].

### 4.6 Contemporary Cardiometabolic Treatment Guidelines

Recent guidelines from the American Heart Association (AHA), American Diabetes Association (ADA) and European Society of Cardiology (ESC) converge on a paradigm shift in the management of diabetes mellitus in the presence of cardiovascular disease. Contemporary recommendations prioritise cardiovascular and renal risk reduction beyond glycaemic control, particularly through the early use of SGLT‐2 inhibitors and GLP‐1 receptor agonists with proven cardiovascular benefit. Table 4 summarises key similarities across these major societies [184, 185, 186, 187].

**TABLE 4:** Key guideline recommendations for diabetes management in the presence of cardiovascular disease.

Recommendation area	Aha (American heart association)	Ada (American diabetes association)	Esc (European society of cardiology)
Risk stratification	Emphasises comprehensive CVD risk assessment in all patients with diabetes; encourages lifetime risk evaluation.	Recommends stratifying patients based on CVD history, risk factors, and presence of subclinical atherosclerosis.	Incorporates risk scoring specific to diabetic patients; encourages early detection of CVD and risk modifier evaluation.
Glycaemic targets	Individualised A1c goal (∼7% or lower if feasible) with tighter control in selected patients without hypoglycaemia risk.	A1c < 7% for most adults; individualisation based on comorbidities and CVD presence.	Similar individualised approach; emphasises avoiding overly strict control in older patients or those at risk for hypoglycaemia.
SGLT‐2 inhibitors	Recommends SGLT‐2 inhibitors as first‐line in patients with T2DM and established CVD or heart failure (HF) unless contraindicated.	Strongly supports use of SGLT‐2 inhibitors with proven CVD benefits (e.g., reduction in HF hospitalisation, renal protection).	Encourages SGLT‐2 inhibitors in diabetes with CVD and/or HF, highlighting heart failure and renal benefit.
GLP‐1 receptor agonists	Advises GLP‐1 receptor agonists with proven CV benefit for patients with T2DM and atherosclerotic CVD.	Recommends GLP‐1 receptor agonists with proven CVD benefit independent of baseline A1c when CVD is present.	Recommends GLP‐1 receptor agonists with established CV benefit as part of comprehensive risk reduction.
Heart failure (HF) focus	Prioritises agents with robust HF outcomes (especially SGLT‐2 inhibitors).	Endorses SGLT‐2 inhibitors for HF with reduced ejection fraction regardless of diabetes status.	Places significant emphasis on SGLT‐2 across HF spectrum; encourages multidisciplinary management.
Renal protection	Highlights SGLT‐2 inhibitors for renal risk reduction in patients with diabetic kidney disease.	Recommends SGLT‐2 inhibitors in patients with CKD (albuminuria or reduced eGFR) for renal and CV protection.	Integrates renal risk prevention into CVD care; recommends SGLT‐2 inhibitors for CKD with cardiovascular implications.
Lifestyle interventions	Encourages heart‐healthy diet, physical activity, weight management, and smoking cessation.	Strong endorsement of lifestyle modification as foundational therapy.	Endorses mediterranean‐like diet, regular exercise, and structured weight loss programs.
Lipid management	Recommends high‐intensity statin therapy for diabetic patients with CVD.	High‐intensity statin therapy for CVD prevention; consider ezetimibe/PCSK9 inhibitors if needed.	Advocates aggressive lipid lowering; considers additional non‐statin therapies to reach low LDL targets in high‐risk patients.
Blood pressure targets	Recommends individualised BP targets, often < 130/80 mmHg for most with diabetes and CVD.	Recommends BP < 130/80 mmHg when tolerable, especially with CVD.	Recommends tailored BP goals; often < 130/80 mmHg in high‐risk patients including diabetics with CVD.
Antiplatelet therapy	Considers low‐dose aspirin in secondary prevention for patients with established CVD.	Recommends aspirin therapy for secondary prevention; individualised for primary prevention after risk evaluation.	Supports low‐dose aspirin for secondary prevention; primary prevention is individualised in high‐risk cases.

Abbreviations: ADA: American Diabetes Association, AHA: American Heart Association, ASCVD: atherosclerotic cardiovascular disease, BP: blood pressure, CKD: chronic kidney disease, eGFR: estimated glomerular filtration rate, ESC: European Society of Cardiology, GLP‐1 RA: glucagon‐like peptide‐1 receptor agonist, HbA1c: glycated haemoglobin, HF: heart failure, HFrEF: heart failure with reduced ejection fraction, LDL: low‐density lipoprotein, PCSK9: proprotein convertase subtilisin/kexin type 9, SGLT‐2: sodium–glucose co‐transporter 2, T2DM: type 2 diabetes mellitus.

## 5 Gut Microbiome in Diabetes and Cardiovascular Diseases

In diabetes mellitus, particularly in advanced stages, vascular dysfunction and peripheral neuropathy can impair tissue perfusion, increasing the susceptibility to ulcers and chronic wounds. Elevated blood glucose concentrations further favour microbial overgrowth and infection, heightening the risk of systemic complications such as bacteraemia, fungaemia, and, in severe cases, sepsis [188].

Beyond localised infections, increasing evidence suggests that gut microbiota dysbiosis plays a critical role in the pathogenesis of both diabetes and cardiovascular disease. The human intestinal microbiota is composed of approximately 10^14^ microorganisms spanning over a thousand species. Disruptions in the diversity and function of these microbial communities have been associated with a variety of chronic diseases, including diabetes mellitus, inflammatory bowel disease, obesity and colorectal cancer [189, 190].

The predominant bacterial phyla in a healthy gut microbiome include Actinobacteria, Proteobacteria, Firmicutes, Bacteroidetes, Verrucomicrobia, and Cyanobacteria [191, 192] In T1D, studies have demonstrated a reduction in beneficial bacteria, such as Akkermansia muciniphila, Bifidobacterium, Prevotella, *Lactobacillus* and Firmicutes, accompanied by an increase in Bacteroidetes and *Clostridium* species [193]. Additionally, maternal exposure to certain viral infections, such as rubella, has been linked to an increased risk of T1D in offspring [194, 195].

Similarly, T2DM is associated with gut microbiota imbalances, characterised by a decline in *Clostridium* species and an increase in *Lactobacillus*, Firmicutes, and *Bacteroides* species. There is also a noted reduction in beneficial butyrate‐producing bacteria, such as Roseburia and Faecalibacterium prausnitzii, alongside an increase in opportunistic pathogens. Although causality remains unclear, this microbial imbalance is believed to disrupt host metabolic homoeostasis and promote systemic inflammation [195].

Cardiovascular diseases are the leading cause of death globally, with multiple contributing factors, including alterations in gut microbiota composition. Dysbiosis has been associated with the development of atherosclerosis, heart failure, and hypertension. Bacterial metabolites, including trimethylamine‐N‐oxide (TMAO), short‐chain fatty acids (SCFAs), and bile acids, have been shown to influence cardiovascular risk by modulating host metabolism, inflammation, and vascular function [196, 197, 198].

Clinical studies have identified specific microbial signatures associated with CVD. For example, increased levels of Collinsella, *Escherichia coli* and *Enterobacter* aerogenes have been observed in atherosclerosis. In patients with coronary artery disease, elevated counts of *Lactobacillus*, *Streptococcus*, *Escherichia*/*Shigella* ratio and *Enterococcus* species have been reported, while beneficial genera such as *Bacteroides*, Faecalibacterium, Prevotella and Roseburia are reduced [199, 200, 201]. Furthermore, microbial analysis of atherosclerotic plaques has revealed an over‐representation of pathogens like *Staphylococcus*, *Proteus* vulgaris, *Streptococcus* and *Klebsiella pneumoniae* [195, 202, 203, 204].

These alterations are associated with inflammatory responses that may exacerbate atherosclerotic progression, although direct causation remains to be established. Specific bacterial metabolites also correlate with disease phenotypes; elevated TMAO levels and reduced SCFA and bile acid production have been linked to increased risks of stroke, mesenteric ischaemia, and peripheral artery disease [205].

Probiotic therapy has been proposed as a potential adjunctive approach to managing diabetes and CVD. Probiotics exert beneficial effects by modulating gut microbiota composition, inhibiting pathogenic bacteria, enhancing immune responses, and improving metabolic profiles. Species commonly used in probiotic formulations include *Streptococcus*, Bifidobacterium, *Lactobacillus* and *Enterococcus*, often administered through fermented foods such as yoghurt and cheese. Emerging data suggest that probiotics may reduce serum cholesterol levels, lower uric acid concentrations, and improve cardiac function, although further research is warranted to define their clinical efficacy [206, 207].

GLP‐1 receptor agonists used in conjunction with metformin have been shown to alter the gut microbiota composition, which may contribute to their therapeutic advantages [208].

## 6 Future Perspectives

Future research is increasingly focused on therapeutic strategies that target the intersecting inflammatory and metabolic pathways underlying both diabetes mellitus and cardiovascular disease. Beyond traditional glucose‐lowering approaches, emerging interventions aim to modulate key inflammatory and oxidative signalling nodes involved in cardiometabolic injury. For example, pharmacological inhibition of the NLRP3 inflammasome and activation of Nrf2‐mediated antioxidant pathways have shown promising effects in experimental models by reducing oxidative stress, myocardial fibrosis, and vascular dysfunction. Similarly, blockade of chemokine signalling pathways such as CCR2/CCR5 may attenuate monocyte recruitment and macrophage‐driven inflammation in cardiometabolic tissues. Another promising avenue involves specialised pro‐resolving mediators (SPMs), which promote the resolution of chronic inflammation rather than simply suppressing inflammatory responses.

In parallel, epigenetic and RNA‐based therapeutic strategies, including modulation of microRNAs and other non‐coding RNAs, are being explored as potential regulators of cardiac remodelling, metabolic dysfunction, and inflammatory signalling in diabetes. In addition, increasing evidence highlights the role of the gut microbiome as a modulator of cardiometabolic disease through the production of metabolites such as trimethylamine‐N‐oxide and short‐chain fatty acids, suggesting that microbiome‐targeted therapies may represent another future therapeutic avenue.

Importantly, these emerging strategies also underscore the need for phenotype‐guided therapeutic approaches. Diabetes‐associated cardiovascular disease represents a heterogeneous spectrum of disorders, and distinct patient phenotypes may be driven by different molecular mechanisms. For instance, obesity‐associated HFpEF is often characterised by systemic inflammation, adipose tissue dysfunction, and metabolic stress, whereas lean individuals with type 1 diabetes may develop cardiovascular complications primarily through microvascular injury and autoimmune‐mediated inflammation. Recognising these mechanistic differences may enable the development of precision‐medicine approaches, in which therapies are tailored according to the dominant molecular pathways present in each patient phenotype [108, 110, 209, 210, 211, 212, 213].

## 7 Conclusion

DM and CVD are closely interconnected conditions driven by a complex network of metabolic, inflammatory and molecular signalling pathways. Persistent hyperglycaemia, insulin resistance and metabolic dysregulation initiate a cascade of pathophysiological processes that contribute to vascular injury, myocardial remodelling and progressive cardiovascular dysfunction. Increasing evidence indicates that these processes do not occur through isolated mechanisms but rather through intersecting molecular pathways that converge on common inflammatory and metabolic signalling hubs.

Among the most important of these shared mechanisms is the NF‐κB–inflammasome axis, which integrates signals from hyperglycaemia, oxidative stress, advanced glycation end products and adipose tissue inflammation to promote chronic low‐grade inflammation and endothelial dysfunction. In parallel, mitochondrial dysfunction and endoplasmic reticulum stress contribute to cardiomyocyte injury, impaired metabolic flexibility and increased oxidative damage in the diabetic myocardium. Additionally, adipose–cardiac cross‐talk, mediated through adipokines and lipid‐derived signalling molecules, plays a critical role in linking systemic metabolic disturbances to myocardial remodelling and vascular dysfunction. Emerging evidence also highlights the contribution of gut microbiome–derived metabolites, which may modulate systemic inflammation, metabolic homoeostasis and atherosclerotic progression.

Importantly, several contemporary cardiometabolic therapies already appear to target elements of these interconnected pathways. For example, SGLT‐2 inhibitors have demonstrated substantial cardiovascular and renal protection, partly through mechanisms that include reductions in oxidative stress, improvements in myocardial energetics, modulation of inflammatory signalling and attenuation of adverse cardiac remodelling. Similarly, GLP‐1 receptor agonists have been associated with reductions in major adverse cardiovascular events, potentially through anti‐inflammatory, anti‐atherosclerotic and metabolic effects. These findings underscore the growing recognition that successful therapies for DM and CVD may exert their benefits not solely through glucose lowering but through modulation of shared molecular pathways underlying cardiometabolic injury.

Future research should continue to focus on identifying and targeting these intersecting mechanisms, including inflammatory signalling networks, mitochondrial and ER stress pathways, adipose tissue–derived mediators and microbiome‐associated metabolic signals. A deeper understanding of these complex interactions may facilitate the development of integrated therapeutic strategies capable of simultaneously addressing metabolic dysfunction and cardiovascular injury. Ultimately, approaches that target shared mechanistic nodes within the cardiometabolic network hold promise for improving outcomes and reducing the global burden of diabetes‐associated cardiovascular disease [190, 194, 207, 214, 215].

## Author Contributions

**Lilian Anagnostopoulou:** conceptualization, methodology, investigation, data curation, writing – original draft, writing – review and editing. **Nikolaos Ktenopoulos:** conceptualization, methodology, investigation, data curation, writing – original draft, writing – review and editing, supervision. **Anastasios Apostolos:** investigation, data curation, writing – original draft. **Christos Fragoulis:** investigation, data curation, writing – original draft. **Panayotis Vlachakis:** investigation, data curation, writing – original draft. **Paschalis Karakasis:** investigation, data curation. **Marios Sagris:** writing – original draft, writing – review and editing. **Nikias Milaras:** writing – review and editing. **Maria Drakopoulou:** writing – review and editing. **Andreas Synetos:** writing – review and editing. **Ioannis Kyriazis:** writing – review and editing. **Ioannis Ioannidis:** writing – review and editing. **Costas Tsioufis:** supervision, writing – review and editing. **Konstantinos Toutouzas:** conceptualization, supervision, writing – review and editing.

## Funding

The authors have nothing to report.

## Ethics Statement

The authors have nothing to report.

## Consent

The authors have nothing to report.

## Conflicts of Interest

The authors declare no conflicts of interest.

## Data Availability

The authors have nothing to report.
